# Resurgence of Dengue Virus Serotype 3 in Minas Gerais, Brazil: A Case Report

**DOI:** 10.3390/pathogens13030202

**Published:** 2024-02-24

**Authors:** Talita Adelino, Maurício Lima, Natália R. Guimarães, Joilson Xavier, Vagner Fonseca, Luiz Marcelo R. Tomé, Maira Alves Pereira, Vanessa Ferreira Machado, Luiz Carlos Junior Alcantara, Felipe C. de Melo Iani, Marta Giovanetti

**Affiliations:** 1 Fundação Ezequiel Dias, Belo Horizonte 30510-010, Brazil; talitadelino@gmail.com (T.A.); maurili15@gmail.com (M.L.); natyroguiman@gmail.com (N.R.G.); lmarcelotome@gmail.com (L.M.R.T.); maira.pereira@funed.mg.gov.br (M.A.P.); 2 Instituto René Rachou, Fundação Oswaldo Cruz, Belo Horizonte 30190-009, Brazilalcantaraluiz42@gmail.com (L.C.J.A.); 3 Department of Exact and Earth Science, University of the State of Bahia, Salvador 41192-010, Brazil; vagner.fonseca@gmail.com; 4 Climate Amplified Diseases and Epidemics (CLIMADE), Brasilia 70070-130, Brazil; 5 Secretaria Municipal de Saúde de Belo Horizonte, Belo Horizonte 30130-012, Brazil; vanessaferreiras@pbh.gov.br; 6 Sciences and Technologies for Sustainable Development and One Health, Università Campus Bio-Medico di Roma, 00128 Roma, Italy

**Keywords:** DENV monitoring, next-generation sequencing, molecular epidemiology

## Abstract

This report provides a detailed overview of the resurgence of DENV-3 in the state of Minas Gerais, Brazil, which is a concerning scenario in the context of dengue, a mosquito-borne viral disease. Historically, Brazil has grappled with dengue epidemics caused primarily by the DENV-1 and DENV-2 serotypes. However, in 2023, a significant shift in this pattern was observed as DENV-3 made a notable resurgence. This resurgence was characterized by the increase in DENV-3 cases within the country and the region of the Americas. Given the absence of sustained DENV-3 circulation in Brazil in previous years, this situation poses a significant risk, making the population highly susceptible to a potential novel epidemic. In November 2023, a 31-year-old male patient in Belo Horizonte exhibited symptoms of acute febrile syndrome. Multiplex RT-qPCR using the Kit Molecular ZC D-Tipagem confirmed DENV-3 infection, suggesting a likely autochthonous case, as the patient reported no travel history. To promptly assess this resurgence, we applied the nanopore sequencing technology. This allowed for the rapid characterization of the initial DENV-3 case isolated in Minas Gerais in 2023, representing a 13-year interval since the serotype’s previous documented circulation in that state. This case report underscores the critical importance of proactive monitoring and the swift implementation of targeted control strategies to address the evolving dynamics of dengue, with a specific emphasis on the resurgence of DENV-3 in the state.

## 1. Introduction

Dengue is a tropical mosquito-borne viral disease caused by dengue viruses (*Orthoflavivirus denguei*, DENV), members of the family *Flaviviridae* and genus *Orthoflavivirus* (formerly named *Flavivirus*) [1]. The main vectors involved in the transmission of this viral pathogen are the *Aedes* mosquitoes, widely distributed in tropical and subtropical regions [2]. DENV is classified into four antigenically distinct and genetically related serotypes (DENV1-4), each containing several genotypes primarily named according to their geographic origin [3]. Since its initial detection in the early 1980s, different serotypes of dengue have been responsible for unexpected and extensive epidemics in Brazil. The country has reported the highest number of cases in the Americas, with approximately 25 million infections up to December 16, 2023 [4]. Although isolated cases of DENV-3 and DENV-4 have been recorded during past epidemics, recent dengue outbreaks in Brazil have primarily been attributed to the circulation of DENV-1 and DENV-2 serotypes [5,6,7,8]. However, there has been a notable shift in this trend in 2023, with the re-emergence of DENV-3, which is currently causing increasing concern. The surge in reports of DENV-3 cases, both in terms of their frequency and geographical distribution across the Americas Region [9,10], is alarming Brazilian health authorities. This situation poses a significant risk of a new epidemic, especially given the historical absence of sustained DENV-3 circulation in the country, leaving the population highly susceptible. Furthermore, the high density of vector mosquitoes combined with increased human mobility, population growth, unplanned urbanization, globalization, and climate change may create favorable conditions for the re-emergence of DENV-3 in Brazil [11]. Up to 13 December 2023, the country has already confirmed a total of 130 cases of DENV-3 across 11 different states.

DENV-3 is classified into five distinct genotypes (I-V), with genotype III (DENV-3 III) recognized as the most widespread, encompassing strains isolated from Asia, Africa, the Caribbean, the Americas, and Europe [12]. Furthermore, DENV-3 III has consistently been associated with severe dengue outbreaks across Asia, Africa, and Latin America. In Brazil, the first case of DENV-3 was reported in December 2000 [13]. In 2002, it was the serotype responsible for a large dengue epidemic in the state of Rio de Janeiro, including 1831 cases of dengue hemorrhagic fever and 91 deaths [14]. Subsequent outbreaks were reported in various regions of Brazil in the following years, underscoring its rapid and extensive spread [9,15].

In Minas Gerais state, situated in the southeastern region and ranking as the second most populous Brazilian state [16], the most recent autochthonous case of DENV-3 was documented in 2010. Here, we promptly describe and characterize the first case of DENV-3 isolated in 2023 in Minas Gerais, marking a 13-year gap without the detected circulation of this serotype in the state.

## 2. Materials and Methods

### 2.1. Sample Collection and Molecular Diagnostic Assay

A serum sample from a male patient suspected of arbovirus infection was sent to the Central Laboratory of Public Health of Minas Gerais (Lacen-MG), located at the Fundação Ezequiel Dias (Funed), as part of routine molecular diagnosis for arboviruses. Viral RNA was extracted using an automated protocol on the MagNA Pure 96 System (Roche, Switzerland) using the MagNA Pure 96 DNA and Viral NA Small Volume Kit (Roche), following the manufacturer’s recommendations. The molecular diagnosis was performed on the LightCycler 480 Instrument II (Roche) using the Kit Molecular ZC D-Tipagem, developed by Bio-Manguinhos/FIOCRUZ (Rio de Janeiro, Brazil) and provided by the Brazilian Ministry of Health (BrMoH) to the public laboratories network. This kit enables the detection and differential diagnosis of arboviruses through RT-qPCR multiplex reactions for DENV serotyping (DENV1-4), Zika virus (*Orthoflavivirus zikaense)*, Chikungunya virus, and the human RNAseP gene, which is used as an endogenous control.

### 2.2. cDNA Synthesis and Whole-Genome Sequencing Using Nanopore Technology 

Viral RNA was subjected to cDNA synthesis using the ProtoScript II First Strand cDNA Synthesis Kit (NEB), following the manufacturer’s recommendations. The resulting cDNA was submitted to sequencing multiplex PCR (35-cycles) using Q5 High Fidelity Hot-Start DNA Polymerase (NEB) and a set of specific primers designed by the CADDE project (https://www.caddecentre.org/ (accessed on 20 November 2023)) for the purpose of sequencing the complete genome of DENV-3. Amplicons were purified using 1× AMPure XP Beads (Beckman Coulter, Brea, CA, USA) and quantified on a Qubit 3.0 fluorimeter (Thermofisher Scientific, Waltham, MA, USA) along with the Qubit™ dsDNA HS Assay Kit (Thermofisher Scientific, United States of America). DNA library preparation was performed using the Ligation Sequencing Kit (SQK-LSK109) and the Native Barcoding Kit (EXP-NBD104) from Oxford Nanopore Technologies [17] and subsequent sequencing on an R9.4 flow cell using the MinION platform (Oxford Nanopore Technologies).

### 2.3. Generation of Consensus Sequence

Raw files were basecalled and demultiplexing using Guppy v.6.0 (Oxford Nanopore Technologies). Consensus sequence was obtained using the Genome Detective online tool (Accessed on 20 November 2023: https://www.genomedetective.com/) [18]. A DENV genotyping assessment was performed using Dengue Virus Typing Tool (https://www.genomedetective.com/app/typingtool/dengue/ (accessed on 20 November 2023)) [19], which was implemented in the Genome Detective platform. The newly generated DENV-3 III sequence was deposited in GISAID under accession number EPI_ISL_18699410.

### 2.4. Phylogenetic Analysis

We constructed phylogenetic trees to explore the relationship of the sequenced genome from Minas Gerais with those of other isolates. We retrieved all complete DENV-3 genomes (n = 1702) with the associated date and country of collection from GenBank and GISAID collected up to December 16, 2023 (Table S1). Sequence alignment was performed using MAFFT v7.3.10 [20] and manually curated to remove artefacts using AliView v1.28 [21]. The GTR nucleotide substitution model, which was inferred as the best-fit model by the ModelFinder application implemented in IQ-TREE2 [22], was used to estimate maximum likelihood phylogenetic trees. The tree topology’s robustness was determined using 1000 bootstrap replicates. We additionally inferred a time-scaled tree by using TreeTime [23].

### 2.5. DENV Epidemiological Data

The number of weekly notified cases of infection by DENV in Brazil from 2014 to 2023 (up to June 2023) was obtained from DATASUS (Unified Health System) and is available for download through PySUS (https://pysus.readthedocs.io/pt/most recent). After downloading, we used these data to calculate the dengue incidence (normalized per 100,000 individuals) in Brazil and the state of Minas Gerais, and to plot a map and time series graph using RStudio 2022.12.0.

## 3. Case Description

On 16 November 2023, a 31-year-old male patient started exhibiting symptoms indicative of acute febrile syndrome, encompassing manifestations such as headache, rash, retroorbital pain, myalgia, nausea, vomiting, arthralgia, diarrhea, and prolonged fever since 18 November 2023. Subsequently, on 21 November 2023, the patient was admitted to a public health unit in Belo Horizonte, the capital of the state of Minas Gerais. On the same day, the patient yielded a positive result on the dengue tourniquet test. Considering the initial suspicion of dengue, a serum sample was promptly collected from the patient and sent to the Lacen-MG for molecular screening. As a routine practice, Lacen-MG subjects all samples from individuals suspected of arboviruses to molecular diagnosis of dengue, zika, and chikungunya. The diagnosis confirmed DENV-3 infection, with a cycle threshold (CT) value of 21. Additionally, the patient resides near a stream area in the northern part of the city; however, he reported being bitten by mosquitoes during a visit to a green area in the eastern part of the city, close to his workplace. Notably, no travel history was reported, suggesting a likely autochthonous case.

**Figure 1:**
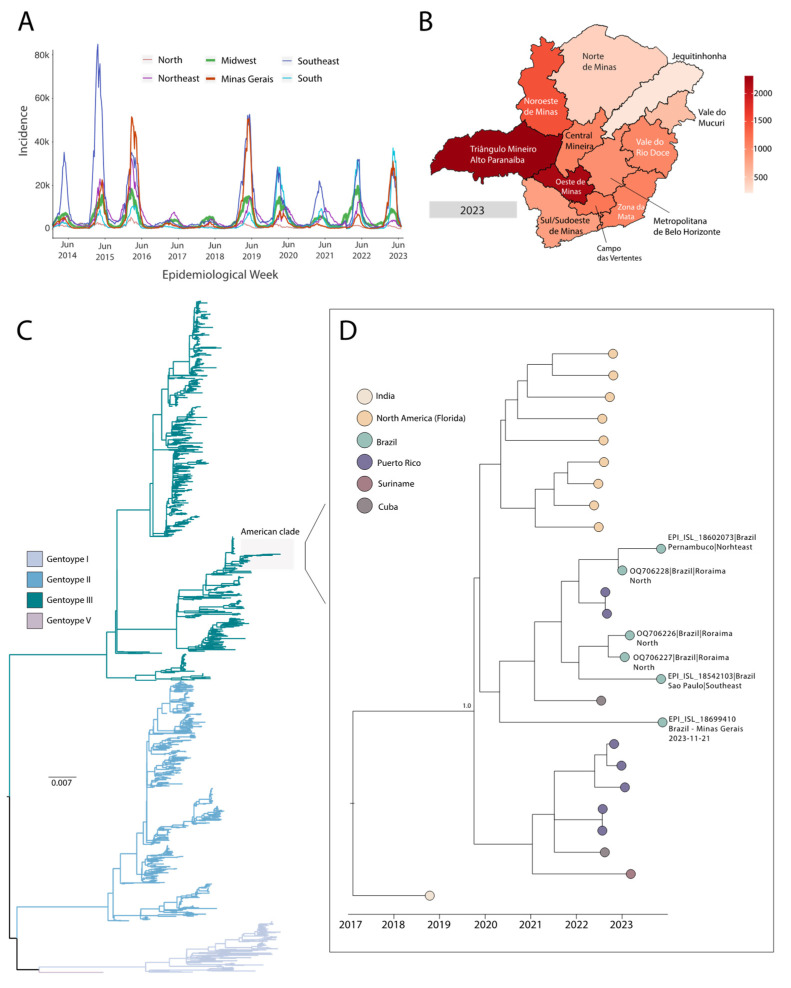
Genomic epidemiology of dengue in Minas Gerais, Brazil. (**A**) Weekly notified dengue cases normalized per 100,000 individuals per Brazilian macro region (North, Midwest, Southeast, Northeast, and South) during 2014–2023 (up to June 2023). Data from the state of Minas Gerais were plotted separately (outside the Southeast macro region) for better visualization. Epidemic curves are colored according to geographical region. (**B**) Weekly notified dengue cases from Minas Gerais state (normalized per 100,000 individuals) divided by health mesoregion during 2023 (up to June 2023). (**C**) Midpoint rooted maximum-likelihood phylogeny of DENV-3 isolates (n = 1702), showing distinct genotypes. The scale bar is in units of substitutions per site (s/s). (**D**) Time-resolved maximum-likelihood tree showing the clade containing our newly generated DENV-3 III sequence from Minas Gerais State, Brazil. Colors represent different sampling locations.

As dengue has been an endemic disease in Brazil, we obtained the number of weekly notified cases from different Brazilian states (aggregated into five Brazilian macro-regions: North, Midwest, Southeast, Northeast, and South) to compare the current epidemiological landscape with those recorded in previous years. The weekly reported incidence (normalized per 100,000 individuals) revealed three major outbreaks across all macro-regions during 2015, 2016, and 2019, with the state of Minas Gerais playing such a prominent role that its reported incidence was higher than that observed in some Brazilian macro-regions (Figure 1A). In contrast, we also observed a significant reduction in the cases between 2017 and 2018 in all macro-regions of Brazil. In 2023, the state of Minas Gerais continued with a high number of dengue cases, standing out as one of the regions with the highest incidences of the disease (Figure 1A). During this period, we observed that the ’Região Metropolitana de Belo Horizonte’, the health mesoregion of Minas Gerais with the first case of DENV-3 confirmed in 2023, had a significant number of reported cases. However, ’Triângulo Mineiro/Alto Paranaíba’ and ’Oeste de Minas’ also emerged as the mesoregions with the highest dengue incidences in the state (Figure 1B).

We performed whole-genome sequencing using MinION technology to rapidly identify the DENV genotype as an integral part of our active real-time monitoring of arboviruses. A total of 64,870 reads were obtained from the DENV-3 genome, with an average depth of 5592× and a coverage of 93.3%. Subsequently, we performed phylogenetic analysis, which classified the genome as DENV-3 genotype III (Figure 1C). Time-resolved maximum-likelihood trees demonstrated that the new isolate from Minas Gerais state clustered with other Brazilian strains isolated in 2023 (Figure 1D). These Brazilian sequences were from different regions across Brazil, including the northeast (Pernambuco, n = 1), north (Roraima, n = 3), and southeast (São Paulo, n = 1). Furthermore, we observed a close relationship of Brazilian sequences with sequences from Puerto Rico (n = 2) and Cuba (n = 1), all of which were sampled in 2022 (Figure 1D). This finding suggests that the Caribbean region might have played a significant role in the dispersion dynamics of DENV-3 III within South American countries, including Brazil.

## 4. Discussion

In this study, we describe the first case of DENV-3 reintroduction in 2023 in the state of Minas Gerais, southeastern Brazil, from a male patient residing in Belo Horizonte, after 13 years since the last autochthonous case recorded in the region. A time series of reported cases between 2014 and 2023 revealed significant epidemic waves in Brazil, especially in 2015, 2016, and 2019. These typical yearly seasonal patterns, as well as a decline in the number of reported cases in 2017 and 2018, possibly reflect alterations in the population’s herd immunity, resulting from exposure to serotype replacement events in recent years [5,24,25,26]. We also noted a significant number of reported cases in the ‘Região Metropolitana de Belo Horizonte’ during 2023. However, even after the confirmation of the first case of DENV-3 in the state, this mesoregion did not emerge as the one with the highest incidence of dengue in Minas Gerais. This observation strongly indicates the necessity of implementing continuous and real-time monitoring practices across all macro-regions.

Phylogenetic analysis has revealed that the newly sequenced DENV-3 genome belongs to genotype III. This finding aligns with previous studies, further confirming that genotype III currently holds its position as the predominant reemerging genotype in the Americas [9,10,12,13,14,27]. Genotype III first made its appearance in the Americas in 1994 during a dengue outbreak in Nicaragua and Panama. It subsequently spread from Central America and Mexico into the Caribbean region and South America [14,28]. Subsequently, DENV-3 III spread into several Brazilian states, and, in 2002, quickly became the predominant serotype in the country [29]. Similarly to the findings in a recent study [9], our DENV-3 III genome clustered with other Brazilian sequences from 2023, as well as with strains from the 2022 Caribbean region [10], suggesting that the Caribbean could be the most likely source of DENV-3 dispersion to Brazil and the Americas. However, the current paucity of complete genome sequences in public databases hampers our understanding of the most likely viral transmission routes.

In Brazil, the last major DENV-3 epidemic was documented in 2008 [30]. Currently, after 15 years without sustained circulation in Brazil and 13 years since the last recorded autochthonous case in Minas Gerais, DENV-3 poses a challenge to our surveillance system due to the risk of a new epidemic in a highly susceptible population. Furthermore, the hyperendemic scenario and the co-circulation of other DENV serotypes emphasize the need to enhance genomic surveillance of dengue in Brazil, especially following the recent approval of the Qdenga vaccine by the BrMoH. With its imminent availability in the Brazilian public healthcare system, it is crucial to reinforce real-time genomic monitoring to uncover mutations linked to immune evasion that might impact the vaccine’s effectiveness.

Currently, Brazil has confirmed 130 cases of DENV-3 in 2023 (up to 13 December 2023), with only seven complete genomes, including our newly generated isolate, which is available in public databases. Although this report describes only a single new case of DENV-3 from the state of Minas Gerais after a significant period, our epidemiological and genomic analyses encompassed all available data, providing valuable insights into the resurgence of this pathogen both regionally and in Brazil. However, it is important to acknowledge the limitations inherent in a single-case study. The findings presented may not fully represent the extent of DENV-3’s spread in Minas Gerais, as there could be further unreported cases. The lack of detection over the past 13 years does not necessarily suggest the true absence of the DENV-3 serotype, particularly considering the possibility of asymptomatic cases or those with mild symptoms that did not seek medical attention or were not correctly diagnosed. Additionally, the circulation of DENV-3 in other regions of Brazil, with movement of people or vectors, may have implications for its presence in Minas Gerais that this single case cannot fully elucidate. This case report, therefore, represents a snapshot of a dynamic and complex epidemiological situation, and further surveillance and research are needed to understand the broader trends of dengue virus serotypes, especially DENV-3, in the state and the country.

In conclusion, our findings confirm the detection of the first DENV-3 in the state of Minas Gerais in 2023 and underscore the critical importance of proactive genomic monitoring as a tool for early detection, swift responses, and effective control measures to protect public health both in Brazil and globally. 

## Data Availability

Newly generated DENV-3 sequences have been deposited into GISAID under accession number EPI_ISL_18699410.

## Supplementary Materials

The following supporting information can be downloaded at: https://www.mdpi.com/article/10.3390/pathogens13030202/s1, Table S1: List of complete reference genomes of DENV-3 available in GenBank and GISAID databases used in this study.
