# Supplementary material for: Resurgence of Dengue Virus Serotype 3 in Minas Gerais, Brazil: A Case Report
Source: Pathogens. 2024 Feb 24;13(3):202. doi: 10.3390/pathogens13030202 (PMC10974589; doi:10.3390/pathogens13030202)
Supplement: Supplementary file 1 [file pathogens-13-00202-s001.zip › pathogens-2849857-supplementary.pdf]

SUPPLEMENTARY MATERIAL

Resurgence and Rapid Detection of Dengue Virus Serotype 3 in Minas Gerais, Brazil: a case report

Table S1. List of complete reference genomes of DENV3 available in GenBank and GISAID databases used in this study.

Accession numbers	Country
EPI_ISL_18542103*	Brazil
EPI_ISL_18602073*	Brazil
MG721059	India
MG721061	India
MG721064	India
KC762681	Indonesia
KC762682	Indonesia
KC762683	Indonesia
KC762684	Indonesia
KC762685	Indonesia
KC762687	Indonesia
KC762686	Indonesia
KC762688	Indonesia
GQ466079	India
KC762690	Indonesia
KC762689	Indonesia
KC762691	Indonesia
KC762692	Indonesia
KC762693	Indonesia
JF920393	Nicaragua
JF920394	Nicaragua
JF920395	Nicaragua
JF920396	Nicaragua
JF920397	Nicaragua
JF920398	Nicaragua
JF920399	Nicaragua
JF920400	Nicaragua
JF920401	Nicaragua
JF920402	Nicaragua
JF920403	Nicaragua

JF920404	Nicaragua
JF920405	Nicaragua
JF920406	Nicaragua
JF920407	Nicaragua
JF920408	Nicaragua
JF920409	Nicaragua
JF937620	Nicaragua
JF937621	Nicaragua
JF937622	Nicaragua
JF937623	Nicaragua
JF937625	Nicaragua
JF937624	Nicaragua
JF937626	Nicaragua
JF937627	Nicaragua
JF937628	Nicaragua
JF937629	Nicaragua
JF937630	Nicaragua
JF937631	Nicaragua
JF937632	Nicaragua
JF937633	Nicaragua
JF937634	Nicaragua
JF937636	Nicaragua
JF937637	Nicaragua
JF937638	Nicaragua
JF937639	Nicaragua
JF937640	Nicaragua
JF937641	Nicaragua
JF937642	Nicaragua
JF937643	Nicaragua
JF937646	Nicaragua
JF937647	Nicaragua
JF937648	Nicaragua
JF937652	Nicaragua
KU216208	India
KU216209	India
GQ868546	Brazil
GQ868547	Brazil
GQ868548	Brazil
GQ868571	Colombia
GQ868572	Colombia
GQ868573	Colombia
GQ868575	Colombia

GQ868574	Colombia
GQ868576	Colombia
GQ868577	Colombia
GQ868578	Colombia
GQ868586	Venezuela
GQ868587	Venezuela
GQ868616	SaintLucia
GQ868617	Trinidad and Tobago
GQ868626	Cambodia
GQ868627	Cambodia
GQ868628	Cambodia
GQ868629	Cambodia
GQ868634	Cambodia
GU131844	Brazil
GU131845	Brazil
GU131846	Brazil
GU131847	Brazil
GU131848	Brazil
GU131849	Brazil
GU131850	Brazil
GU131851	Brazil
GU131852	Brazil
GU131853	Brazil
GU131854	Brazil
GU131855	Brazil
GU131856	Brazil
GU131857	Brazil
GU131858	Brazil
GU131859	Brazil
GU131860	Brazil
GU131861	Brazil
GU131862	Brazil
GU131865	Brazil
GU131866	Brazil
GU131867	Brazil
GU131868	Brazil
GU131869	Brazil
GU131871	Brazil
GU131870	Brazil
GU131872	Brazil
GU131873	Brazil
GU131874	Brazil

GU131875	Brazil
GU131876	Brazil
GU131877	Brazil
GU131878	Brazil
GU131903	Cambodia
GU131904	Cambodia
GU131905	Cambodia
GU131906	Cambodia
GU131907	Cambodia
GU131908	Cambodia
GU131909	Cambodia
GU131910	Cambodia
GU131911	Cambodia
GU131912	Cambodia
GU131913	Cambodia
GU131914	Cambodia
GU131915	Cambodia
GU131916	Cambodia
GU131917	Cambodia
GU131918	Cambodia
GU131933	Cambodia
GU131934	Cambodia
GU131935	Cambodia
GU131936	Cambodia
GU131937	Cambodia
GU131938	Cambodia
GU131939	Cambodia
GU131940	Cambodia
GU131941	Cambodia
GU131942	Cambodia
GU131943	Cambodia
GU131944	Cambodia
GU131945	Cambodia
GU131946	Cambodia
GU131950	Colombia
GU131951	Colombia
GU131952	Colombia
GU131953	Colombia
GU131954	Colombia
DQ401689	Bangladesh
DQ401691	Bangladesh
DQ401692	Bangladesh

DQ401693	Bangladesh
DQ675521	Taiwan
DQ675520	Indonesia
DQ675522	Taiwan
DQ675523	Taiwan
DQ675524	Taiwan
DQ675525	Taiwan
DQ675526	Taiwan
DQ675527	Taiwan
DQ675528	Taiwan
DQ675529	Taiwan
DQ675530	Taiwan
DQ675531	Taiwan
DQ675532	Taiwan
DQ675533	Taiwan
MW288025	Senegal
MW288026	Senegal
FJ205870	USA
FJ205871	USA
KU509278	Barbados
FJ373302	USA
FJ373303	Venezuela
FJ373304	Venezuela
FJ373306	USA
KU509281	India
KU509282	Senegal
KU509283	SriLanka
MW288027	Senegal
KU509284	Thailand
KU509285	Thailand
KU509286	India
MW288028	Senegal
FJ390372	USA
FJ390377	USA
FJ410178	USA
HM181934	Cambodia
HM181935	Cambodia
FJ432728	Vietnam
FJ432731	Vietnam
FJ432741	Vietnam
FJ432743	Vietnam
HM181972	Nicaragua

HM181973	Nicaragua
HM181974	Nicaragua
HM181975	Nicaragua
FJ461322	Vietnam
FJ461337	Vietnam
FJ461338	Vietnam
EU081181	Singapore
EU081183	Singapore
EU081186	Singapore
EU081187	Singapore
EU081188	Singapore
EU081191	Singapore
EU081192	Singapore
EU081196	Singapore
EU081202	Singapore
EU081203	Singapore
EU081207	Singapore
EU081208	Singapore
EU081209	Singapore
EU081211	Singapore
MW288031	Senegal
EU081215	Singapore
EU081219	Singapore
EU081220	Singapore
EU081221	Singapore
EU081199	Singapore
EU081223	Singapore
EU081225	Singapore
FJ744700	Venezuela
FJ547066	Vietnam
FJ744726	Thailand
FJ547070	USA
FJ547071	USA
FJ547073	USA
FJ547076	USA
FJ547080	USA
FJ547081	USA
FJ744727	Thailand
FJ547083	USA
JN000936	Nicaragua
FJ562097	Vietnam
FJ562099	Vietnam

JN000938	Nicaragua
FJ562103	Vietnam
MW288033	Senegal
FJ744728	Thailand
FJ744729	Thailand
FJ744730	Thailand
FJ744731	Thailand
FJ744732	Thailand
FJ744733	Thailand
FJ744734	Thailand
FJ639713	Cambodia
FJ639714	Cambodia
FJ639715	Cambodia
FJ744735	Thailand
FJ639719	Cambodia
FJ639721	Cambodia
FJ639723	Cambodia
FJ639725	Cambodia
FJ639726	Cambodia
FJ639727	Cambodia
FJ639728	Cambodia
FJ639729	Cambodia
FJ639730	Cambodia
FJ639749	Venezuela
FJ744736	Thailand
FJ639753	Venezuela
FJ639755	Venezuela
FJ639756	Venezuela
FJ639757	Venezuela
FJ639759	Venezuela
FJ639763	Venezuela
FJ639765	Venezuela
FJ639767	Venezuela
FJ639770	Venezuela
FJ639772	Venezuela
FJ744737	Thailand
FJ639778	Venezuela
FJ639779	Venezuela
FJ639781	Venezuela
FJ639785	Venezuela
MW288035	Senegal
FJ744738	Thailand

KJ737429	Thailand
KJ737430	Thailand
FJ639786	Venezuela
FJ639790	Venezuela
FJ639792	Venezuela
FJ744740	Thailand
FJ639798	Venezuela
FJ639801	Venezuela
FJ639803	Venezuela
FJ639807	Venezuela
FJ639810	Venezuela
FJ744739	Thailand
FJ639816	Venezuela
FJ639817	Venezuela
FJ639825	Venezuela
FJ639826	Venezuela
FJ639827	Venezuela
JN093513	Nicaragua
JN093514	Nicaragua
FJ687448	Thailand
JN183884	Nicaragua
JX669489	Brazil
JX669490	Brazil
JX669491	Brazil
AB189125	Indonesia
JX669493	Brazil
JX669495	Brazil
JX669497	Brazil
JX669498	Brazil
JX669499	Brazil
JX669501	Brazil
JX669502	Brazil
MW288037	Senegal
AB189127	Indonesia
JX669503	Brazil
JX669504	Brazil
JX669505	Brazil
KF921914	Nicaragua
KF921916	Nicaragua
JX669508	Brazil
KF921917	Nicaragua
AB189126	Indonesia

KF921919	Nicaragua
KF921920	Nicaragua
KF921924	Nicaragua
KF921927	Nicaragua
KF921928	Nicaragua
AB189128	Indonesia
JN406515	Australia
KF954945	China
KF954946	China
KF954949	China
HM631854	Cambodia
KF955332	Cambodia
KF955333	Cambodia
MW288038	Senegal
HM631856	Nicaragua
HM631858	Nicaragua
HM631857	Nicaragua
KF955453	Venezuela
KF955456	Puerto Rico
KF955457	Vietnam
KF955458	Vietnam
KF955462	Cambodia
KF955465	Puerto Rico
HM631859	Nicaragua
KF955467	Puerto Rico
KF955468	Puerto Rico
KF955471	Venezuela
KF955472	Venezuela
KF955473	Brazil
KF955474	Sri Lanka
HM631860	Nicaragua
KF955479	Venezuela
KF955487	Venezuela
KF955488	Nicaragua
KF955489	Nicaragua
KF955490	Nicaragua
HM631861	Nicaragua
KF955505	Grenada
HM631862	Nicaragua
KF955506	Cambodia
KF955508	Cambodia
KF971696	Nicaragua

KF971700	Nicaragua
KF971705	Nicaragua
HM631863	Nicaragua
KF971708	Nicaragua
KF971711	Nicaragua
KF971695	Nicaragua
HM631864	Nicaragua
KF971704	Nicaragua
KF973476	Nicaragua
KF973477	Nicaragua
KF973478	Nicaragua
KF973481	Nicaragua
HM631869	Nicaragua
KF973482	Nicaragua
KF973487	Nicaragua
MW288039	Senegal
EU482453	Vietnam
EU482454	Vietnam
EU482455	Vietnam
EU482457	Vietnam
EU482456	Vietnam
EU482555	USA
EU482563	USA
EU482564	USA
EU482566	USA
EU482596	USA
EU482612	Venezuela
EU482613	Venezuela
GU363549	China
KJ189255	Peru
KJ189257	Peru
KJ189258	Peru
KJ189259	Peru
KJ189260	Peru
KJ189261	Peru
KJ189262	Peru
KJ189263	Peru
KJ189264	Peru
KJ189266	Peru
KJ189267	Peru
KJ189270	Peru
KJ189273	Peru

KJ189274	Peru
KJ189275	Peru
KJ189276	Peru
KJ189278	Peru
KJ189279	Peru
KJ189280	Peru
KJ189281	Peru
KJ189282	Peru
KJ189283	Peru
KJ189284	Peru
KJ189285	Peru
JN662391	China
KJ189287	Peru
KJ189288	Peru
KJ189290	Peru
KJ189291	Peru
KJ189294	Peru
KJ189295	Peru
MW288040	Senegal
KJ189297	Peru
KJ189298	Peru
KJ189299	Peru
KJ189301	Peru
EU529683	Venezuela
EU529685	Venezuela
EU529686	Venezuela
EU529687	Venezuela
EU529690	Venezuela
EU529691	Venezuela
EU529692	USA
EU529696	USA
EU529697	USA
EU529698	USA
EU529702	USA
EU529704	USA
EU529705	USA
EU569688	Venezuela
EU569689	Venezuela
EU596492	USA
EU596493	USA
EU596494	USA
AB214879	Timor Leste

HG316483	Thailand
HG316484	Thailand
AB214880	Timor Leste
AB214882	Timor Leste
EU660408	Vietnam
EU660410	Vietnam
EU660411	Vietnam
AB214881	Timor Leste
EU660420	Venezuela
MW295815	China
GU370052	Singapore
GU370053	Singapore
EU687196	USA
EU687197	USA
EU687198	USA
EU687233	USA
EU687234	USA
EU687239	USA
JQ045690	Vietnam
JQ045691	Vietnam
JQ045692	Vietnam
JQ045693	Vietnam
JQ045694	Vietnam
JQ045695	Vietnam
AY496871	Bangladesh
AY496873	Bangladesh
EU726769	USA
EU726773	USA
EU726774	USA
EU781136	USA
AY496874	Bangladesh
EU781137	USA
AY496877	Bangladesh
EU854292	Venezuela
EU854291	Venezuela
EU854298	USA
EU932687	Venezuela
EU932688	Venezuela
MW308179	Mexico
AY099336	Sri Lanka
AY099337	Martinique
JQ922556	India

JQ922557	India
KJ622191	China
KJ622192	China
KJ622193	China
KJ622194	China
MW308180	Mexico
KJ622195	China
KJ622196	China
KJ622197	China
KJ622198	China
KJ622199	China
KJ643590	Peru
FJ024465	USA
FJ024466	USA
FJ024467	USA
FJ024468	USA
FJ024469	USA
FJ024470	USA
FJ024471	USA
FJ177308	Brazil
FJ182004	USA
FJ182005	USA
FJ182006	USA
FJ182007	USA
FJ182008	USA
FJ182009	USA
FJ182010	USA
FJ182011	USA
FJ182013	USA
FJ182015	Venezuela
FJ182037	USA
FJ182038	USA
FJ182039	USA
FJ182040	USA
FJ182041	USA
MW308181	Mexico
MF004386	Malaysia
MF142763	Thailand
LC379193	Gabon
LC379194	Gabon
LC379195	Gabon
LC379196	Gabon

HM756274	Nicaragua
LC379197	Gabon
NC_001475	Sri Lanka
HM756275	Nicaragua
HM756276	Nicaragua
HM756277	Nicaragua
HM756278	Nicaragua
HM756279	Nicaragua
HM756280	Nicaragua
LC410192	Thailand
LC410193	Thailand
LC410194	Thailand
LC410195	Thailand
HM756281	Nicaragua
HM756282	Nicaragua
LC436676	Bangladesh
LC436677	Bangladesh
FJ810413	Thailand
FJ810414	Thailand
FJ810416	Venezuela
FJ850048	Nicaragua
FJ850049	Nicaragua
FJ850052	Nicaragua
FJ850055	USA
FJ850056	USA
FJ850079	Brazil
MN922033	China
MN922034	China
FJ850080	Brazil
MN922035	China
MN922036	China
MN922039	China
MN922040	China
MN922041	China
FJ850083	Brazil
FJ850086	Brazil
FJ850089	Brazil
FJ850092	Brazil
MN964273	China
FJ850094	Brazil
MN964274	China
MT006152	Sri Lanka

MT006169	Sri Lanka
MH544647	Colombia
MH544649	Colombia
MH544650	Colombia
MH544651	Colombia
FJ850096	Venezuela
KU509280	Thailand
FJ850097	Venezuela
FJ850098	Venezuela
MK858148	India
MK858149	India
MK858150	India
MK858151	India
MK858152	India
MK858153	India
MK858154	India
MK858155	India
MK894338	China
MK894339	China
MK894340	China
MK894341	China
MF370226	China
MT261972	Burkina Faso
MT261973	Burkina Faso
MT261974	Burkina Faso
MT261975	Burkina Faso
MT261976	Burkina Faso
MT261977	Burkina Faso
MT261978	Burkina Faso
MT261979	Burkina Faso
MN018367	China
MN018368	China
MN018369	China
MN018370	China
MN018371	China
MN018372	China
MN018373	China
MN018374	China
MN018375	China
MN018376	China
MN018377	China
MN018378	China

MN018381	China
MN018382	China
MN018383	China
MN018384	China
MN018385	China
MN018386	China
MN018387	China
MN018388	China
FJ850109	Venezuela
FJ850110	Venezuela
MF682966	China
MF682967	China
MF682968	China
MF682969	China
MF682970	China
MF682971	China
MF682972	China
MF682973	China
MF682974	China
MF682975	China
FJ850111	Venezuela
KF041255	Pakistan
MN083245	Sri Lanka
MN083246	Sri Lanka
KF041254	Pakistan
KF041256	Pakistan
MK005258	Malaysia
KF041257	Pakistan
LT898451	Malaysia
LT898452	Malaysia
KF041258	Pakistan
KF041259	Pakistan
HQ166030	Nicaragua
HQ166031	Nicaragua
HQ166032	Nicaragua
KY670634	Taiwan
HQ166033	Nicaragua
HQ166034	Nicaragua
MT921575	Australia
MH822957	India
MH823209	Indonesia
KY794786	Papua New Guinea

KY794787	Papua New Guinea
KY794788	Papua New Guinea
KY794789	Papua New Guinea
KY794790	Papua New Guinea
KR296743	China
KR296744	China
FJ873812	Nicaragua
FJ873813	Nicaragua
FJ882571	Sri Lanka
FJ882573	Sri Lanka
KY849761	Laos
KY849769	Laos
KY849770	Laos
KY849771	Laos
KY849772	Laos
KY849773	Laos
KY849774	Laos
KY849775	Laos
FJ882575	Mozambique
FJ882576	Nicaragua
FJ882577	Venezuela
FJ882578	Venezuela
KX380839	Singapore
KX380840	Singapore
KX380841	Singapore
KX380842	Singapore
MH888332	Thailand
KY863456	Indonesia
MH888333	Bolivia
MH891766	India
KY921906	Singapore
KY921907	Singapore
KT424097	Thailand
MN227697	China
MN227698	China
MN227699	China
MN227700	China
MN227701	China
MN227702	China
MN227703	China
HQ235027	Paraguay
FJ898440	Mexico

LT996904	Malaysia
LT996905	Malaysia
LT996906	Malaysia
LT996907	Malaysia
LT996908	Malaysia
LT996909	Malaysia
LT996910	Malaysia
LT996911	Malaysia
LT996912	Malaysia
FJ898441	Mexico
MN253124	India
MN253125	India
MN253126	India
FJ898442	Mexico
MN253127	India
MN253128	India
MN253129	India
MN253130	India
MN253131	India
MN253132	India
MN253133	India
FJ898443	Colombia
FJ898444	Colombia
FJ898445	Colombia
FJ898446	Brazil
FJ898447	Brazil
FJ898457	Ecuador
KT726340	Cuba
KT726341	Cuba
FJ898458	Peru
KT726342	Cuba
KT726343	Cuba
KT726344	Cuba
KT726345	Cuba
KT726346	Cuba
KT726347	Cuba
KT726348	Cuba
KT726349	Cuba
KT726350	Cuba
KT726351	Cuba
FJ898459	Trinidad and Tobago
KT726352	Cuba

KT726353	Cuba
KT726354	Cuba
KT726355	Cuba
KT726356	Cuba
KT726357	Cuba
KT726358	Cuba
KT726359	Cuba
KT726360	Cuba
KT726361	Cuba
FJ898462	Anguilla
FJ898463	Saint Lucia
FJ898464	Guyana
KX855927	India
FJ898468	Venezuela
FJ898469	Venezuela
FJ898470	Venezuela
FJ898471	Venezuela
FJ898472	Venezuela
MH048677	Malaysia
FJ898473	Venezuela
MH051731	Malaysia
MH051732	Malaysia
MH051733	Malaysia
FJ898474	Venezuela
MN448930	Thailand
MN448931	Thailand
MN448932	Thailand
MN448933	Thailand
MN448934	Thailand
MN448935	Thailand
MN448936	Thailand
MN448937	Thailand
MN448938	Thailand
MN448939	Thailand
FJ898475	Nicaragua
MN448940	Thailand
MN448941	Thailand
MN448942	Thailand
MN448943	Thailand
MN448944	Thailand
MN448945	Thailand
MN448946	Thailand

MN448947	Thailand
MN448948	Thailand
MN448949	Thailand
FJ898476	Nicaragua
MN448950	Thailand
MN448951	Thailand
MN448952	Thailand
MN448953	Thailand
MN448954	Thailand
MN448955	Thailand
MN448956	Thailand
MN448957	Thailand
MN448958	Thailand
MN448959	Thailand
FJ913015	Brazil
MN448960	Thailand
MN448961	Thailand
MN448962	Thailand
MN448963	Thailand
MN448964	Thailand
MN448965	Thailand
MN448966	Thailand
MN448967	Thailand
MN448968	Thailand
MN448969	Thailand
HQ332170	Venezuela
MN448970	Thailand
MN448971	Thailand
MN448972	Thailand
MN448973	Thailand
MN448974	Thailand
MN448975	Thailand
MN448976	Thailand
MN448977	Thailand
MN448978	Thailand
HQ332171	Venezuela
MN448979	Thailand
MN448980	Thailand
MN448981	Thailand
MN448982	Thailand
MN448983	Thailand
MN448984	Thailand

MN448985	Thailand
MN448986	Thailand
MN448987	Thailand
MN448988	Thailand
MN448989	Thailand
MN448990	Thailand
MN448991	Thailand
MN448992	Thailand
MN448993	Thailand
MN453624	Singapore
HQ541785	Nicaragua
HQ541789	Nicaragua
HQ541790	Nicaragua
HQ541791	Nicaragua
HQ541795	Nicaragua
HQ541796	Nicaragua
HQ541797	Nicaragua
HQ541802	Nicaragua
HQ541803	Nicaragua
HQ541804	Nicaragua
HQ541806	Nicaragua
KF973486	Nicaragua
KF973485	Nicaragua
KF973484	Nicaragua
KF973483	Nicaragua
KF973480	Nicaragua
KF973479	Nicaragua
KF971710	Nicaragua
KF971709	Nicaragua
KF971703	Nicaragua
KF971702	Nicaragua
KF971699	Nicaragua
JX669507	Brazil
JX669506	Brazil
JX669500	Brazil
JX669496	Brazil
JX669494	Brazil
JX669492	Brazil
KF954948	China
JN406514	Australia
KF954947	China
KJ830751	Saudi Arabia

KJ189300	Peru
KJ189296	Peru
KJ189293	Peru
KJ189292	Peru
KJ189289	Peru
KJ189286	Peru
KJ189277	Peru
KJ189272	Peru
KJ189271	Peru
KJ189269	Peru
AY662691	Singapore
KJ189268	Peru
KJ189265	Peru
KJ189256	Peru
KF824903	China
KF824902	China
AY676348	Thailand
AY676350	Thailand
JQ411814	Sri Lanka
AY676349	Thailand
KF955507	Cambodia
KF955486	Venezuela
KF955481	Venezuela
KF955480	Venezuela
AY676351	Thailand
KF955466	Puerto Rico
KF955464	Cambodia
KF955463	Cambodia
KF955461	Cambodia
KF955460	Vietnam
KF955459	Vietnam
KF955454	Venezuela
KF955451	Venezuela
KF955449	Venezuela
AY676352	Thailand
KF955335	Nicaragua
EU081224	Singapore
EU081222	Singapore
EU081218	Singapore
EU081217	Singapore
EU081216	Singapore
EU081214	Singapore

EU081213	Singapore
EU081212	Singapore
AY676353	Thailand
EU081210	Singapore
EU081206	Singapore
EU081205	Singapore
EU081204	Singapore
EU081201	Singapore
EU081200	Singapore
EU081198	Singapore
EU081197	Singapore
KF921929	Nicaragua
KF921926	Nicaragua
KF921925	Nicaragua
EU081195	Singapore
EU081194	Singapore
KF921923	Nicaragua
EU081193	Singapore
EU081190	Singapore
KF921922	Nicaragua
KF921921	Nicaragua
KF921913	Nicaragua
EU081189	Singapore
EU081185	Singapore
EU081184	Singapore
EU081182	Singapore
KC425219	Brazil
KC425218	Brazil
KC261634	China
JQ045689	Vietnam
JQ045688	Vietnam
JQ045687	Vietnam
JN368477	Cambodia
JN093517	Nicaragua
JN093515	Nicaragua
JN000937	Nicaragua
HM181978	Nicaragua
HM181977	Nicaragua
HM181976	Nicaragua
HM181933	Cambodia
FJ639731	Cambodia
FJ639724	Cambodia

FJ639722	Cambodia
FJ639720	Cambodia
FJ639716	Cambodia
FJ639712	Cambodia
FJ461334	Vietnam
FJ461329	Vietnam
FJ461326	Vietnam
FJ432722	Vietnam
FJ639805	Venezuela
FJ639804	Venezuela
FJ639800	Venezuela
FJ639799	Venezuela
FJ639795	Venezuela
FJ639793	Venezuela
FJ639791	Venezuela
FJ639789	Venezuela
FJ639787	Venezuela
FJ639784	Venezuela
FJ639782	Venezuela
FJ639780	Venezuela
FJ639777	Venezuela
FJ639776	Venezuela
FJ639775	Venezuela
FJ639774	Venezuela
FJ639771	Venezuela
FJ639769	Venezuela
FJ639768	Venezuela
FJ639766	Venezuela
FJ639762	Venezuela
FJ639761	Venezuela
FJ639760	Venezuela
FJ639758	Venezuela
FJ639754	Venezuela
FJ639752	Venezuela
FJ639751	Venezuela
FJ639750	Venezuela
FJ639747	Venezuela
FJ639746	Venezuela
FJ562107	USA
FJ562102	Vietnam
FJ562100	Vietnam
FJ547085	USA

FJ547084	USA
FJ547082	USA
FJ547079	USA
FJ547078	USA
FJ547077	USA
FJ547075	USA
FJ547074	USA
FJ547072	USA
FJ547069	USA
FJ547062	Vietnam
FJ547061	Vietnam
FJ478456	USA
EU367962	China
FJ410229	Vietnam
FJ410177	USA
FJ410176	USA
FJ390376	USA
FJ390375	USA
FJ390373	USA
FJ390371	USA
EU726772	USA
EU726771	USA
EU726768	USA
EU687226	USA
EU687221	USA
EU687219	USA
EU687218	USA
EU660409	Vietnam
EU660407	Vietnam
EU569691	Venezuela
EU569690	Venezuela
EU529703	USA
EU529699	USA
EU529689	Venezuela
EU529688	Venezuela
EU529684	Venezuela
FJ644564	India
GU189648	China
EU482614	Venezuela
EU482595	USA
EU482559	USA
EU482558	USA

EU482462	Vietnam
EU482461	Vietnam
EU482460	Vietnam
EU482459	Vietnam
EU482458	Vietnam
EU482452	Vietnam
MZ008478	Nicaragua
MZ008477	Nicaragua
MZ008476	Nicaragua
MZ008475	Nicaragua
MZ008474	Nicaragua
MZ008473	Nicaragua
MZ008472	Nicaragua
MZ008471	Nicaragua
MZ008470	Nicaragua
MZ008469	Nicaragua
MZ008468	Nicaragua
MW945430	Vietnam
MW945429	Vietnam
MW945428	Puerto Rico
MW946985	Thailand
MW946984	Thailand
MW946983	Thailand
MW946982	Thailand
MW946981	Thailand
MW946980	Thailand
MW946979	Thailand
MW946978	Thailand
MW946977	Thailand
MW946976	Thailand
MW946975	Thailand
MW946974	Thailand
MW946973	Thailand
MW946972	Myanmar
MW946971	Thailand
MW946970	Thailand
MW946969	Thailand
MW946968	Thailand
MW946967	Thailand
MW946966	Thailand
MW946965	Thailand
MW946964	Thailand

MW946963	Thailand
MW946962	Thailand
MW946961	Thailand
MW946960	Thailand
MW946959	Thailand
MW946958	Thailand
MW946957	Thailand
MW946956	Thailand
MW946954	Thailand
MW946953	Thailand
MW946952	Thailand
MW946951	Thailand
MW946950	Thailand
MW946949	Thailand
MW946948	Thailand
MW946947	Thailand
MW946946	Thailand
MW946945	Thailand
MW946944	Thailand
MW946943	Thailand
MW946942	Thailand
MW946941	Thailand
MW946940	Thailand
MW946939	Thailand
MW946938	Thailand
MW946937	Thailand
MW946936	Thailand
MW946935	Thailand
MW946934	Thailand
MW946933	Thailand
MW946932	Thailand
MW946931	Thailand
MW946930	Thailand
MW946929	Thailand
MW946928	Thailand
MW946927	Thailand
MW946926	Thailand
MW946925	Thailand
MW946924	Thailand
MW946923	Thailand
MW946922	Thailand
MW946921	Thailand

MW946920	Thailand
MW946919	Thailand
MW946918	Nicaragua
MW946917	Thailand
MW946916	Thailand
MW946915	Thailand
MW946914	Thailand
MW946913	Thailand
MW946912	Thailand
MW946911	Thailand
MW946910	Thailand
MW946909	Thailand
MW946908	Thailand
MW946906	Thailand
MW946907	Thailand
MW946905	Thailand
MW946904	Thailand
MW946902	Thailand
MW946903	Thailand
MW946901	Thailand
MW946900	Thailand
MW946899	Thailand
MW946898	Thailand
MW946897	Thailand
MW946896	Thailand
MW946895	Thailand
MW946894	Thailand
MW946893	Thailand
MW946892	Thailand
MW946891	Thailand
MW946890	Thailand
MW946889	Thailand
MW946888	Thailand
MW946887	Thailand
MW946886	Thailand
MW946885	Thailand
MW946884	Thailand
MW946883	Thailand
MW946882	Thailand
MW946881	Thailand
MW946880	Thailand
MW946879	Thailand

MW946878	Thailand
MW946877	Thailand
MW946876	Thailand
MW946875	Thailand
MW946874	Thailand
MW946873	Thailand
MW946872	Thailand
MW946871	Thailand
MW946870	Thailand
MW946869	Thailand
MW946868	Thailand
MW946867	Thailand
MW946866	Thailand
MW946865	Thailand
MW946864	Thailand
MW946863	Thailand
MW946862	Thailand
MW946861	Thailand
MW946860	Thailand
MW946859	Thailand
MW946858	Thailand
MW946857	Thailand
MW946856	Thailand
MW946855	Thailand
MW946854	Thailand
MW946853	Thailand
MW946852	Thailand
MW946851	Thailand
MW946850	Thailand
MW946849	Thailand
MW946848	Thailand
MW946847	Thailand
MW946846	Thailand
MW946845	Thailand
MW946844	Thailand
MW946842	Thailand
MW946843	Thailand
MW946841	Thailand
MW946840	Thailand
MW946838	Thailand
MW946839	Thailand
MW946837	Thailand

MW946836	Thailand
MW946835	Thailand
MW946834	Thailand
MW946833	Thailand
MW946832	Thailand
MW946831	Thailand
MW946830	Thailand
MW946829	Thailand
MW946828	Thailand
MW946827	Thailand
MW946826	Thailand
MW946825	Thailand
MW946824	Thailand
MW946823	Thailand
MW946822	Thailand
MW946821	Thailand
MW946820	Thailand
MW946819	Thailand
MW946817	Thailand
MW946818	Thailand
MW946816	Thailand
MW946815	Thailand
MW946814	Thailand
MW946813	Thailand
MW946812	Thailand
MW946811	Thailand
MW946810	Thailand
MW946809	Thailand
MW946808	Thailand
MW946807	Thailand
MW946806	Thailand
MW946805	Thailand
MW946804	Thailand
MW946803	Thailand
MW946802	Thailand
MW946801	Thailand
MW946800	Thailand
MW946797	Thailand
MW946798	Thailand
MW946799	Thailand
MW946796	Thailand
MW946795	Thailand

MW946792	Thailand
MW946794	Thailand
MW946793	Thailand
MW946791	Thailand
MW946790	Thailand
MW946789	Thailand
MW946788	Thailand
MW946787	Thailand
MW946786	Thailand
MW946785	Thailand
MW946784	Thailand
MW946783	Thailand
MW946782	Thailand
MW946781	Thailand
MW946780	Thailand
MW946779	Thailand
MW946777	Thailand
MW946776	Thailand
MW946778	Thailand
MW946775	Thailand
MW946774	Thailand
MW946773	Thailand
MW946772	Thailand
MW946771	Thailand
MW946770	Thailand
MW946769	Thailand
MW946768	Thailand
MW946767	Thailand
MW946766	Thailand
MW946765	Thailand
MW946764	Thailand
MW946763	Thailand
MW946762	Thailand
MW946761	Thailand
MW946760	Thailand
MW946759	Thailand
MW946758	Thailand
MW946757	Thailand
MW946756	Thailand
MW946755	Thailand
MW946754	Thailand
MW946753	Thailand

MW946752	Thailand
MW946751	Thailand
MW946750	Thailand
MW946749	Thailand
MW946748	Thailand
MW946747	Thailand
MW946746	Thailand
MW946745	Thailand
MW946744	Thailand
MW946743	Thailand
MW946742	Thailand
MW946741	Thailand
MW946740	Thailand
MW946739	Thailand
MW946738	Thailand
MW946737	Thailand
MW946736	Thailand
MW946735	Thailand
MW946734	Thailand
MW946733	Thailand
MW946732	Thailand
MW946731	Thailand
MW946730	Thailand
MW946729	Thailand
MW946728	Thailand
MW946727	Thailand
MW946726	Thailand
MW946725	Thailand
MW946724	Thailand
MW946723	Thailand
MW946722	Thailand
MW946721	Thailand
MW946720	Thailand
MW946719	Thailand
MW946718	Thailand
MW946717	Thailand
MW946716	Thailand
MW946715	Thailand
MW946714	Thailand
MW946713	Thailand
MW946712	Thailand
MW946711	Thailand

MW946710	Thailand
MW946709	Thailand
MW946708	Thailand
MW946707	Thailand
MW946706	Thailand
MW946705	Thailand
MW946704	Thailand
MW946703	Thailand
MW946702	Thailand
MW946701	Thailand
MW946700	Thailand
MW946699	Thailand
MW946698	Thailand
MW946697	Thailand
MW946696	Thailand
MW946695	Thailand
MW946694	Thailand
MW946693	Thailand
MW946692	Thailand
MW946690	Thailand
MW946689	Thailand
MW946688	Thailand
MW946687	Thailand
MW946686	Thailand
MW946685	Thailand
MW946684	Thailand
MW946683	Thailand
MW946682	Thailand
MW946681	Thailand
MW946680	Thailand
MW946679	Thailand
MW946678	Thailand
MW946677	Thailand
MW946676	Thailand
MW946675	Thailand
MW946674	Thailand
MW946673	Thailand
MW946671	Thailand
MW946672	Thailand
MW946670	Thailand
MW946669	Thailand
MW946668	Thailand

MW946667	Thailand
MW946666	Thailand
MW946665	Thailand
MW946664	Thailand
MW946663	Thailand
MW946662	Thailand
MW946661	Thailand
MW946660	Thailand
MW946659	Thailand
MW946658	Thailand
MW946657	Thailand
MW946656	Thailand
MW946655	Thailand
MW946654	Thailand
MW946653	Thailand
MW946652	Thailand
MW946651	Thailand
MW946650	Thailand
MW946649	Thailand
MW946648	Thailand
MW946647	Thailand
MW946646	Thailand
MW946645	Thailand
MW946644	Thailand
MW946643	Thailand
MW946642	Thailand
MW946641	Thailand
MW946640	Thailand
MW946639	Thailand
MW946638	Thailand
MW946637	Thailand
MW946636	Thailand
MW946635	Thailand
MW946634	Thailand
MW946633	Thailand
MW946632	Thailand
MW946631	Thailand
MW946630	Thailand
MW946629	Thailand
MW946628	Thailand
MW946627	Thailand
MW946626	Thailand

MW946625	Thailand
MW946624	Thailand
MW946623	Thailand
MW946622	Thailand
MW946621	Thailand
MW946620	Thailand
MW946619	Thailand
MW946618	Thailand
MW946617	Thailand
MW946616	Thailand
MW946614	Thailand
MW946615	Thailand
MW946613	Thailand
MW946612	Thailand
MW946610	Thailand
MW946611	Thailand
MW946609	Thailand
MW946608	Thailand
MW946607	Thailand
MW426463	China
MW192824	India
MW192823	India
MW192822	India
MW192821	India
MW192820	India
AY679147	Brazil
OM368353	China
MZ857204	Kenya
MZ857217	Saudi Arabia
MZ857225	Thailand
MZ857226	Thailand
MW788878	Myanmar
MW788877	Myanmar
MW788881	Myanmar
MW788879	Myanmar
MW788880	Myanmar
MW788882	Myanmar
MW788883	Myanmar
MW788886	Myanmar
MW788885	Myanmar
MW788884	Myanmar
MW788887	Myanmar

MW788888	Myanmar
MW788890	Myanmar
MW788889	Myanmar
MW788892	Myanmar
MW788891	Myanmar
MW788894	Myanmar
MW788893	Myanmar
MW788895	Myanmar
MW788896	Myanmar
MW788897	Myanmar
MW788898	Myanmar
MW788899	Myanmar
MW788900	Myanmar
MW788901	Myanmar
MW788903	Myanmar
MW788902	Myanmar
MW788906	Myanmar
MW788905	Myanmar
MW788904	Myanmar
MW788908	Myanmar
MW788907	Myanmar
MW788909	Myanmar
MW788910	Myanmar
MW788911	Myanmar
MW788912	Myanmar
OM417340	Mexico
OM417341	Mexico
OK598123	Sri Lanka
OK598124	Sri Lanka
OK598125	Sri Lanka
OK605762	Paraguay
OK605763	Indonesia
OK605764	Thailand
OK605765	Ecuador
OK605766	Somalia
OK631736	Sri Lanka
MZ312921	India
ON007080	Thailand
ON055565	Thailand
ON055566	Thailand
ON055567	Thailand
ON055568	Thailand

ON109599	India
ON123655	India
ON123658	India
ON123660	India
ON123659	India
ON123662	India
ON123665	India
ON123669	India
ON123670	India
HQ671176	Nicaragua
HQ671177	Nicaragua
MZ544585	Kenya
MZ544586	Kenya
MZ544587	Kenya
MZ544588	Kenya
HQ671178	Nicaragua
OK469353	Thailand
OK469354	Thailand
HQ705609	Nicaragua
OM638675	India
MW720883	China
MW720884	China
MW720886	China
MW720885	China
HQ705610	Nicaragua
MW720888	China
MW720887	China
MW720889	China
MW720890	China
HQ705611	Nicaragua
HQ705612	Nicaragua
HQ705613	Nicaragua
HQ705614	Nicaragua
HQ705615	Nicaragua
HQ705616	Nicaragua
HQ705617	Nicaragua
ON799401	India
ON890788	Ethiopia
ON890789	Maldives
ON890819	China
ON890832	China
ON891145	China

ON900159	China
HQ705618	Nicaragua
ON907582	China
ON907583	Bangladesh
ON908232	Ethiopia
ON908231	Bangladesh
ON908233	Maldives
ON908234	China
ON908235	Bangladesh
ON908236	China
ON908237	China
ON908238	Thailand
HQ705619	Nicaragua
ON908245	China
HQ705620	Nicaragua
HQ705621	Nicaragua
HQ705622	Nicaragua
HQ705623	Nicaragua
HQ891025	Nicaragua
OP410998	Singapore
OP410997	Singapore
OP410996	Singapore
OP410993	Singapore
OP410994	Singapore
AY770511	India
MW396463	Bangladesh
MW396464	Bangladesh
MW396462	Bangladesh
MW396469	Bangladesh
MW396467	Bangladesh
MW396465	Bangladesh
MW396468	Bangladesh
MW396466	Bangladesh
OP895705	India
OP895929	Maldives
OP895928	Maldives
OP921002	India
OQ103092	Sri Lanka
OQ103113	Sri Lanka
OQ103114	Sri Lanka
OQ103123	Sri Lanka
OQ103317	Sri Lanka

OQ445908	USA
OQ445911	USA
OQ445934	USA
OQ445946	USA
OQ445929	USA
OQ445932	USA
OQ445961	USA
OQ445962	USA
OQ445957	USA
OM865777	Bhutan
OM865778	Bhutan
OM865779	Bhutan
OM865780	Bhutan
OM865782	Bhutan
OM865781	Bhutan
OM865783	Bhutan
OM865784	Bhutan
OM865786	Bhutan
OM865785	Bhutan
OM865787	Bhutan
OM865788	Bhutan
OM865790	Bhutan
OM865789	Bhutan
OM865791	Bhutan
OM865792	Bhutan
OM865793	Bhutan
OM865794	Bhutan
OM865795	Bhutan
OM865797	Bhutan
OM865796	Bhutan
OM865798	Bhutan
OM865799	Bhutan
OM865801	Bhutan
OM865800	Bhutan
OM865802	Bhutan
OM865804	Bhutan
OM865803	Bhutan
OM865805	Bhutan
OM865806	Bhutan
OM865808	Bhutan
OM865807	Bhutan
OM865809	Bhutan

OM865810	Bhutan
OM865811	Bhutan
OM865812	Bhutan
OM865813	Bhutan
OM865814	Bhutan
OM865815	Bhutan
OM865818	Bhutan
OM865817	Bhutan
OM865816	Bhutan
OM865819	Bhutan
OM865820	Bhutan
OQ721955	India
OQ721956	India
OQ721957	India
OQ721958	India
OQ721959	India
OQ721961	India
OQ721960	India
OQ721962	India
OQ721963	India
OQ727062	Brazil
OQ747070	Cuba
OQ747071	Cuba
AY858037	Indonesia
AY858038	Indonesia
AY858039	Indonesia
AY858040	Indonesia
AY858041	Indonesia
AY858042	Indonesia
AY858043	Indonesia
AY858044	Indonesia
AY858045	Indonesia
AY858046	Indonesia
AY858047	Indonesia
AY858048	Indonesia
AY876494	Thailand
AY923865	Thailand
AF317645	China
GQ199860	Nicaragua
GQ199861	Nicaragua
GQ199862	Nicaragua
GQ199863	Nicaragua

GQ199864	Nicaragua
GQ199865	Nicaragua
GQ199870	Nicaragua
GQ199871	Nicaragua
GQ199886	Nicaragua
GQ199891	Colombia
GQ252674	Sri Lanka
GQ252678	Venezuela
JF295012	Cambodia
JF504679	China
JF808118	Brazil
JF808119	Brazil
JF808120	Brazil
JF808121	Brazil
JF808122	Paraguay
JF808123	Paraguay
JF808124	Brazil
JF808125	Brazil
JF808126	Brazil
JF808127	Brazil
JF808128	Paraguay
JF808129	Paraguay
OQ706226	Brazil
OQ706227	Brazil
OQ706228	Brazil
OQ868517	Brazil
OQ339138	India
OQ836205	Puerto Rico
OQ836206	Puerto Rico
OQ836207	Puerto Rico
OQ836208	Puerto Rico
OQ836209	Puerto Rico
OQ836210	Puerto Rico
OQ836211	Puerto Rico

*GISAID_ID
